# Improving Layer Adhesion of Co-Extruded Polymer Sheets by Inducing Interfacial Flow Instabilities

**DOI:** 10.3390/polym14030587

**Published:** 2022-01-31

**Authors:** Raffael Rathner, Claudia Leimhofer, Wolfgang Roland, Alexander Hammer, Bernhard Löw-Baselli, Georg Steinbichler, Sabine Hild

**Affiliations:** 1 Institute of Polymer Processing and Digital Transformation, Johannes Kepler University Linz, Altenberger Str. 69, 4040 Linz, Austria; raffael.rathner@jku.at (R.R.); alexander.hammer@jku.at (A.H.); bernhard.loew-baselli@jku.at (B.L.-B.); georg.steinbichler@jku.at (G.S.); 2 Pro2Future GmbH, Altenberger Str. 69, 4040 Linz, Austria; 3 Institute of Polymer Science, Johannes Kepler University Linz, Altenberger Str. 69, 4040 Linz, Austria; claudia.leimhofer_1@jku.at (C.L.); sabine.hild@jku.at (S.H.)

**Keywords:** polymer processing, layer adhesion, multi-layer structure, confocal Raman microscopy, flow instabilities, interfaces

## Abstract

Co-extrusion is commonly used to produce polymer multilayer products with different materials tailoring the property profiles. Adhesion between the individual layers is crucial to the overall performance of the final structure. Layer adhesion is determined by the compatibility of the polymers at the interface and their interaction forces, causing for example the formation of adhesive or chemical bonds or an interdiffusion layer. Additionally, the processing conditions, such as temperature, residence time, cooling rate, and interfacial shear stress, have a major influence on the interactions and hence resulting layer adhesion. Influences of temperature and residence time are already quite well studied, but influence of shear load on the formation of an adhesion layer is less explored and controversially discussed in existing literature. In this work, we investigated the influence of different processing conditions causing various shear loads on layer adhesion for a two-layer co-extruded polymer sheet using a polypropylene and polypropylene talc compound system. Therefore, we varied the flow rates and the flow geometry of the die. Under specific conditions interfacial flow instabilities are triggered that form micro layers in the transition regime between the two layers causing a major increase in layer adhesion. This structure was analyzed using confocal Raman microscopy. Making use of these interfacial flow instabilities in a controlled way enables completely new opportunities and potentials for multi-layer products.

## 1. Introduction

Co-extrusion is a process in which two or more polymers are plasticated in different extruders and then joined together in a die. The two die systems that are most common in co-extrusion for flow combination are multi-manifold dies and feedblock systems, which have their advantages and disadvantages [1]. Multi-manifold dies combine the melt streams shortly prior to exiting the die, which enables processing of polymer melts with larger differences in viscosity and melt temperature. On the other hand, in feedblock systems, the individual melt streams are combined in an adapter and conveyed to a monolayer die for final shaping [2]. The major advantage of feedblock systems is their significantly higher flexibility regarding number of layers and their build-up.

Using one of these co-extrusion processing techniques, single products–mainly sheets, profiles, pipes, fibres, and films–with two or more layers are formed. Thus, various materials with different physicochemical properties can be combined to obtain a product with superior properties. For instance, one can embed post-industrial and/or post-consumer recycling material, combine opaque and transparent materials with high surface gloss, provide barrier properties, and improve the mechanical properties of a product. Apart from that, sufficient adhesion between the individual layers to avoid delamination within the service life of a product is essential.

Adhesion between polymers has been studied intensively by various scientific disciplines. The mechanisms considered responsibly for adhesion are categorized typically into [3,4]: (i) mechanical interlocking (lock and key effect), (ii) molecular bonding (covering dipole–dipole interactions, van der Waals forces, and chemical bonds), and (iii) thermodynamic mechanisms (interfacial equilibrium due to minimization of the surface free energy). The mechanism mainly determining delamination strength between layers is, at first, resulting from the material combination under consideration. Systems that are immiscible or partially miscible are typically bonded by using additional tie layers. Tie layers are resins containing functional groups that form bonds (e.g., covalent bonds) with their adjacent materials. In comparison, the diffusion of miscible polymers into each other is much more pronounced, creating an interphase–a region of finite thickness with varying concentration from one bulk phase to the other. Interdiffusion was first proposed by Voyutskii and Vakula [5] and its rate (and hence layer adhesion) was found to be mainly affected by the materials’ molecular weight, polarity, structure (sterical hindrance, cross-linking), and phase state.

Apart from material properties, adhesion between compatible and incompatible polymers in co-extrusion is significantly governed by processing conditions. For instance, increasing process temperatures (melt and die) and reducing the cooling capacity of the downstream calibration and cooling equipment typically increases the adhesion strength. Elevated temperatures amplify chemical bonding and enhance molecular mobility for interdiffusion [4,6,7,8]. Additionally, increasing contact times (by increasing die land and reducing overall throughputs) during co-extrusion also results in longer times for these mechanisms to occur [4,5,6,7,8]. It is further reported that layer adhesion is affected by a high level of shrinkage of one of the materials to be joined [4], and thus shear stresses acting at the interface within the die. Kim and Han [9] introduced an orientation factor that reduces interdiffusion coefficients to model the effect of shear load on interdiffusion using Fick´s laws. On the other hand, Lamnawar et al. [10] suggested that local viscosity imbalances at the interface favour local convective mixing and, consequentially, promote interdiffusion and adhesion.

In this work, we investigated the interplay between die geometry, processing condition, and layer adhesion for bi-layer polypropylene-based sheets that were co-extruded in a feedblock-type die system. To this end, we varied the interfacial residence time and shear load by adjusting the overall throughput and the position of the restrictor bar within the die, respectively. Layer adhesion was then measured by a floating roller peel test, revealing that longer contact times result in higher layer adhesion for the given material combination. Furthermore, narrowing the flow-channel by adjusting the restrictor bar led to a significant increase in layer adhesion. Confocal Raman spectroscopy of the samples revealed that at the restriction bar region, flow instabilities were induced that subsequently led to the formation of a multi-layered structure at the interface during the flow in the coathanger die.

## 2. Materials and Methods

### 2.1. Materials

In this study, a virgin polypropylene homo polymer (polymer) (PP BE50) from the company Borealis and a polypropylene talc compound (compound) with 47w% talc were used. The compound is produced via a co-rotating twin-screw extruder and additionally contains 10w% in-house recyclate. The melt flow rate (MFR), melt density at 230 °C, and bulk density of the two materials are listed in Table 1.

**Figure 1:**
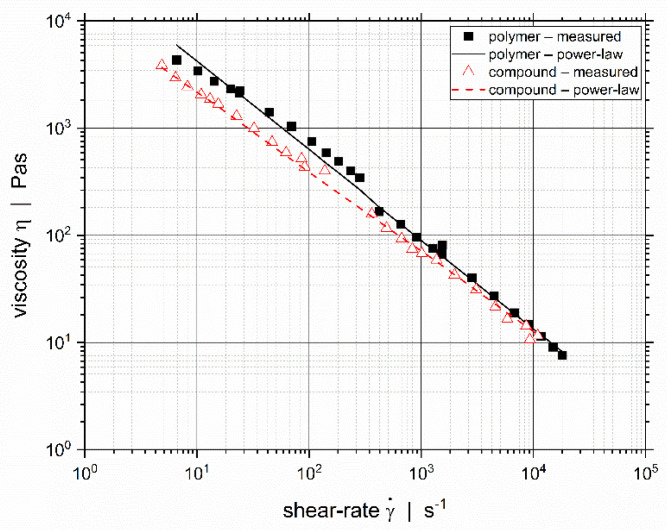
Measured shear-rate dependent viscosity data and power-law approximation for the temperature setting of 235 °C.

The shear viscosity was measured using a Thermo Haake Rheomex slit-die extrusion rheometer (ThermoFisher Scientific, Waltham, MA, USA). The used slit die has two different gaps with h=2 mm and h=0.5 mm, respectively for lower and higher shear-rates [11], and the pressure drop is measured individually for each shear region. The melt is provided by a lab-scale 19/33D single-screw extruder (ThermoFisher Scientific, Waltham, MA, USA). At the screw tip a melt pump and bypass valve are mounted. The melt pump controls the volumetric melt flow-rate through the measurement slit and remaining material exits through the bypass. The viscosity measurements were conducted for two temperature settings: 205 °C and 235 °C. The exact melt temperature was measured by a melt temperature sensor right before the slit entry of the rheometer die. We applied the Weissenberg–Rabinowitsch correction to the measurement data according to [12]. Next, we approximated the shear-rate dependent viscosity η by applying the temperature-dependent power-law model (Equation (1)), with the consistency at the reference temperature $K_{0}$, the power-law exponent n, the reference temperature $T_{0}$, and the temperature sensitivity coefficient β. The derived power-law parameters are listed in Table 2, and a comparison between the measured and the approximated viscosity data is given in Figure 1 for the temperature setting of 235 °C. Note that the temperature of each individual measurement point depicted may vary, which is considered by the temperature dependency of the power-law model.

(1) $$\eta =K_{0}\;e^{-\beta \;\left(T-T_{0}\right)}\;{\left|\dot{\gamma }\right|}^{n-1}.$$

### 2.2. Co-Extrusion

#### 2.2.1. Equipment

**Figure 2:**
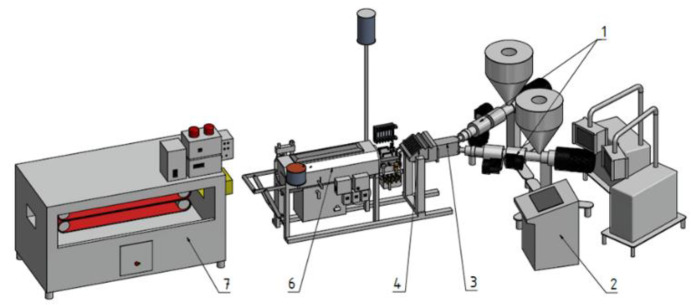
Schematic representation of the co-extrusion line used: 1. extruders; 2. temperature control box; 3. feeding block; 4. co-extrusion die; 5. vacuum calibration and water-cooling tank; 6. haul-off device.

For the co-extrusion experiments, we used a two-layer co-extrusion line, as schematically shown in Figure 2. The combination of the two melt streams within a fixed-flow-divider-type feedblock is followed by shaping of the sheet geometry within a coathanger-type slot die (EMO, Micheldorf, Austria). Upon exiting the extrusion die, the co-extrudate is moved through the downstream vacuum calibration unit where the melt is cooled and the final shape is fixed. Further cooling is done in a spray water cooling tank and, finally, the sheet passes a belt haul-off unit which pulls it through the down-stream line.

**Figure 3:**
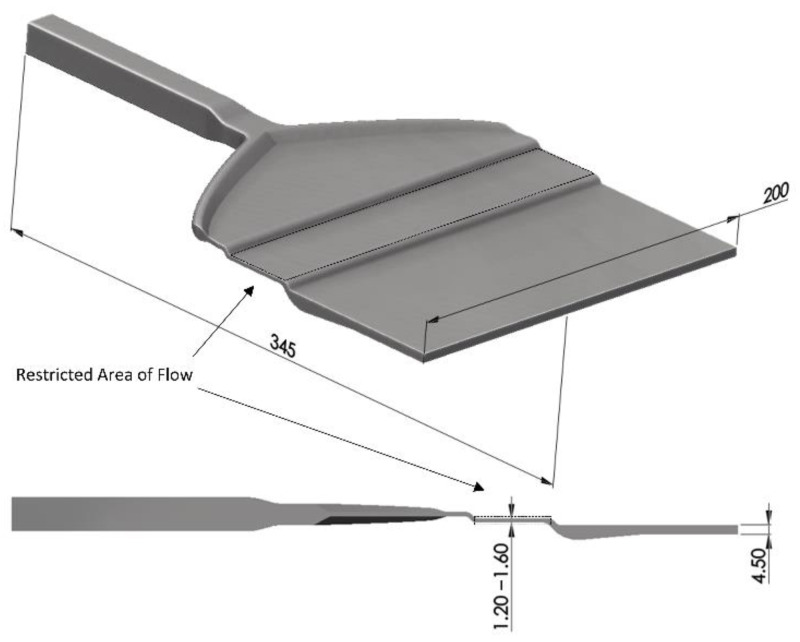
Schematic representation of the three-dimensional flow domain and cross-section of the coathanger manifold.

The die system represents the key component of the sheet co-extrusion line, schematically illustrated in Figure 3. The overall flow domain and the cross-section of the coathanger die is shown in Figure 3. In the restricted area of flow, the flow domain that is marked by the dashed area, the channel height can be manipulated by a restriction bar between 1.6 mm and 1.2 mm, subsequently influencing the flow speed at this position. Consequentially, the processing window of the die can be widened through obtaining different flow properties (e.g., shear rates, shear stresses, and viscosities).

The materials were plasticated using two smooth-barrel, single-screw extruders ECE-Co-Extruder-30 (extrunet, Eberstalzell, Austria) having a diameter of D=30 mm and an axial length of $L=606\;\mathrm{mm}\;\left(L=20.2\;D\right)$. Furthermore, single-flighted, square-pitched three-zone screws with a compression ratio of 2.32 were used. To determine melt temperature and back pressure of the die, each extruder was equipped with a melt temperature sensor and a pressure transducer directly after the screw tip. The extruder barrels, the adapter system that connected extruder and feedblock, and the die system were heated electrically using the temperature profile given in Table 3.

#### 2.2.2. Screw Characteristics

**Figure 4:**
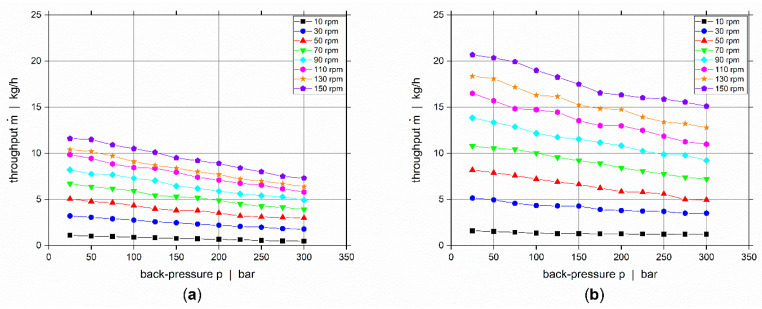
Screw characteristic curves for (**a**) polymer, and (**b**) compound.

For controlling the flow rates of each individual layer in the co-extrusion flow, the screw characteristic curves have to be determined prior to the co-extrusion experiments. Both ECE co-extruders are equipped with identical screws and they have a smooth feed intake section. Consequently, the screw characteristic curves will be back-pressure dependent. Hence, we used an adjustable back-pressure valve for determining the extruder output as a function of screw speed and back pressure. The extruder temperatures are according to the temperature settings for the co-extrusion experiments, listed in Table 3. Screw speed and back-pressure spectrums are varied to cover all expected processing conditions as listed in Table 4 and the back-pressure valve is adjusted accordingly for each screw speed. This represents a two-parametric full-factorial design study with eight and twelve levels, respectively for screw speed and back-pressure, resulting in 96 different operating points for the extruder’s throughput rate. For each throughput measurement, the mean value was created based on three repetitions. The measured screw characteristic curves for the polymer and compound are depicted in Figure 4 for various screw speeds showing that the throughput decreases with increasing back-pressure.

The measured screw-characteristic curves, enable control of the flow rates of each individual layer and hence the layer thicknesses, depending on the back-pressure of the co-extrusion die. Based on these data sets, we developed symbolic regression models for the extruder throughput $\dot{m}$ as a function of screw speed N and back-pressure p, expressing the measured behavior within a simple mathematical function that can easily be implemented into any expert system [13]. Furthermore, and most importantly, it enables easy interpolation within the whole parameter space scanned experimentally. Hence, accurate predictions for operating points that lie on and between the grid points of the full factorial design study, that will occur in the co-extrusion experiments, are easily possible by the use of the obtained mathematical relationships. For deriving the symbolic regression models, we used the open-source software package HeuristicLab [14], which builds on evolutionary algorithms based on genetic programming [15]. The data was therefore randomly split into a training and a test partition, respectively covering 58 and 38 independent samples. The model complexity was restricted by limiting the maximum tree length with 25, and limiting the function set to additions, multiplications, and subtractions. The obtained symbolic regression models for the polymer and compound are given by Equations (2) and (3), respectively. Both throughput models are very simple in their structure with eight and seven constants listed in Table 5 for the polymer and in Table 6 for the compound, respectively.

(2) $$\begin{array}{ll} {\dot{m}}_{polymer}=a_{0}\;+ & a_{1}\;p+a_{2}\;N\\ & +\left(1+a_{3}\;p+a_{4}\;N\right)\left(1+a_{5}\;p+a_{6}\;N\right)\left(a_{7}\;p+a_{8}\;N\right); \end{array}$$

(3) $${\dot{m}}_{compound}=b_{0}+N\left(b_{1}+b_{2}\;p\right)+N^{2}\left(b_{3}+b_{4}\;p\right)+N^{2}\;p^{2}\left(b_{5}\;N+b_{6}\;p+b_{7}\right).$$

**Figure 5:**
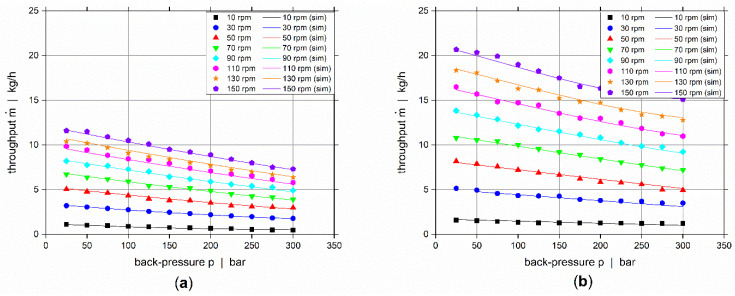
Screw characteristics: comparison of regression models with the experimental data for (**a**) polymer, and (**b**) compound. Experimental data is depicted by the points, and the approximation by the regression models using lines.

The error analysis performed confirms the high accuracy of the developed regression models for the screw characteristics with the statistical measures listed in Table 7. A direct comparison between the experimental data and the predictions using the regression models is given in Figure 5.

#### 2.2.3. Processing Conditions

In order to obtain samples that give reproducible results in layer adhesion testing, the layer distribution was adjusted accurately. The overall sheet with a thickness of 4 mm is composed out of a 3 mm thick layer of the compound and a 1 mm thick layer out of the polymer. To investigate the influence of residence time within the die, three different overall throughputs—given in Table 8—were selected. The individual screw rotational speeds necessary for the given throughputs were adjusted using the symbolic regression models and the melt pressure transducer at the screw tip. This requires an iterative procedure, since the throughputs are dependent on the back-pressure of the die. The mean residence time $\bar{t}$ in co-extrusion was calculated as follows:

(4) $$\bar{t}=\frac{V}{\dot{V}},$$

with the volume of the flow domain V and the total volumetric flow rate $\dot{V}$. Furthermore, the restriction bar was adjusted to two different heights to investigate its influence on flow properties, and consequentially, layer adhesion. For samples 1 to 3 the channel height in the restricted flow area $h_{restrict}$ was set to 1.6 mm, whereas for samples 4 to 6 it was lowered to 1.2 mm.

## 3. Adhesion Experiments

**Figure 6:**
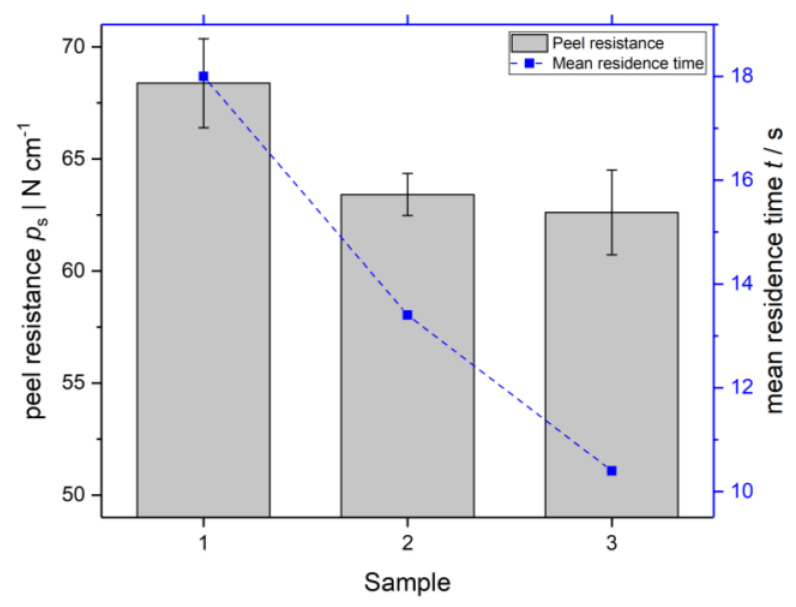
Measured peel resistance of samples 1, 2 and 3 (N=4) and the respective mean residence time in the die.

Adhesion testing was performed using the roller peel test based on DIN EN 1464 [16]. For this purpose, the co-extruded sheets were cut into test specimens with a size of 25 mm × 200 mm, as defined by the EN standard. An initial delamination at the interface is necessary in order to position the sample in the tensile testing device. The peel resistance was measured under a constant test speed of 400 mm min^−1^ and each measurement was repeated four times (N=4). The peel resistance $p_{s}$ is calculated according to Equation (5) with the mean peel force $\bar{F}$ and the sample width w. The results of the roller peel test and the respective mean residence times in the die of samples 1 to 3 are illustrated in Figure 6.

(5) $$p_{s}=\frac{\bar{F}}{w}.$$

In correlation to the literature [17], the peel resistance declines with decreasing residence time for samples 1 to 3. With increasing residence time, the extent of interdiffusion and interlayer entanglement enhances, strengthening the adhesion at the interface. For samples 4 to 6 the roller peel test could not be applied. Initial delamination at the interface, necessary to perform the test, was not possible due to high adhesion.

## 4. Spectroscopic Analysis

To investigate the cause of the high interlayer adhesion in samples 4–6, the interface of the co-extruded sheets was analyzed using confocal Raman microscopy. Raman spectroscopy is a powerful tool to characterize the interface of two polymers that have been joined in the molten state [18] which is based on the inelastic interaction of laser light upon irradiation of sample molecules. The combination of a Raman spectrometer with an optical microscope allows spectral imaging with a resolution of approximately 1 µm. 

### 4.1. Raman Imaging and Set-Up 

To prepare a smooth surface for the subsequent measurement, the samples were cut perpendicular to the interface at –80 °C using a UCT microtome by Leica (Vienna, Austria) equipped with a glass knife. For Raman imaging, an Alpha300R confocal Raman microscope by WITec (Ulm, Germany) coupled with an Nd:YAG laser (λ=532 nm) was used. The laser power was set to 15 mW with integration times ranging from 0.5 to 1 s. The measurements were performed using an Epiplan 20× (0.4, ∞/0) objective by Zeiss (Jena, Germany), which resulted in spot sizes of 1.6 µm. A spectral resolution of approximately 3 cm^−1^ was achieved by using a diffraction grating with 600 grooves per cm. Spectral processing was done using the software Project 5.3 by WITec [19]. The images were analyzed according to the relative area of the CH_2_-/CH_3_-streching vibration bands of polypropylene ($\tilde{\nu }=2900\;{\mathrm{cm}}^{-1}$) using a false-color scale.

### 4.2. Interface of the Co-Extruded Samples

**Figure 7:**
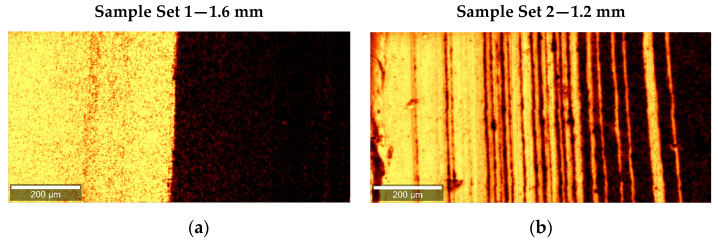
Raman image of the interface between the polymer (yellow) and the compound (black) (**a**) before and (**b**) after the adjustment of the restriction bar.

To investigate the reason for the highly different results of the adhesion experiment, the interfaces of all samples were analyzed by means of confocal Raman microscopy. Characteristic interface images for samples 1 to 3, as well as for samples 4 to 6 are presented in Figure 7.

A color scale relative to the area of the investigated Raman bands is used to represent the different layers of the co-extruded sheets. The yellow domains show the distribution of the polymer, while the dark regions indicate the presence of the compound. Before adjustment of the restriction bar, a smooth interface between the layers was observed. Upon changing the channel height to 1.2 mm, a multi-layer structure formed, as shown in Figure 7, resulting in numerous alternating layers consisting of the individual materials. The multiplication of the layers causes the formation of additional interfaces and explains the failure of the initial delamination in the roller peel tests. 

### 4.3. Investigation of the Development of a Multilayer in the Flow Domain

**Figure 8:**
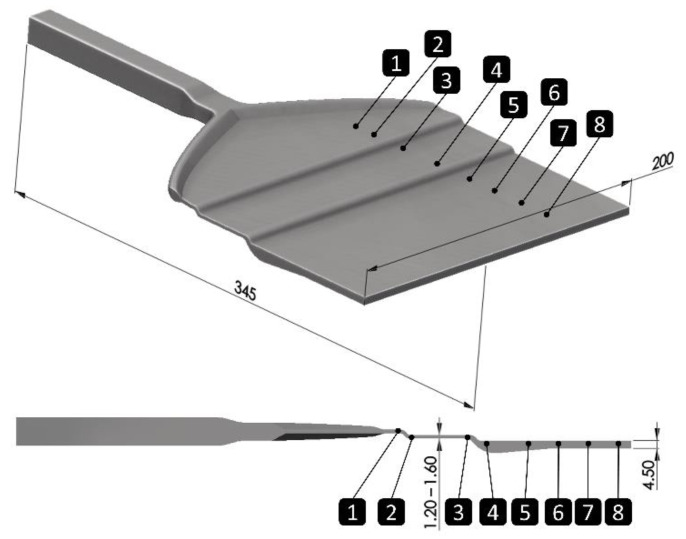
Schematic representation of the melt imprint indicating sample positions 1 to 8 for spectroscopic analysis via confocal Raman microscopy.

**Figure 9:**
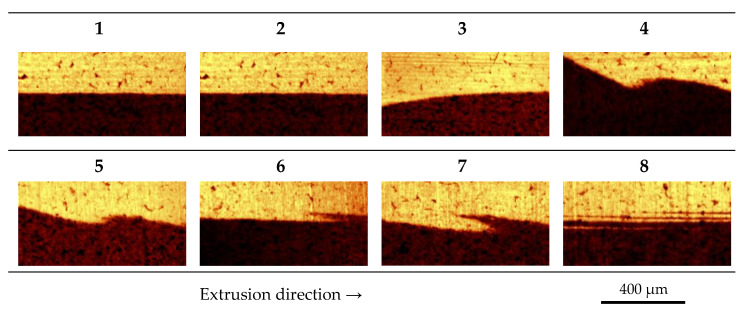
Raman images (400 × 800 µm, 101 × 201 spectra) at the different positions 1 to 8 of the melt imprint.

By setting the extrusion parameters to those used for sample 5, the development of the multilayer formation was investigated in more detail with the aid of a melt imprint. For this purpose, the whole co-extrusion line was started and as soon as steady-state processing conditions were observed the co-extrusion line was abruptly stopped and cooled to room temperature. Subsequently, the die was re-heated to 100 °C enabling opening of the die and detachment of the melt imprint. From the received melt imprint at the specified positions shown in Figure 8, samples were cut to investigate the development of the interface. To determine how and where the formation of the multilayer takes place in the die, we analyzed these probes from the melt imprint by means of confocal Raman microscopy. The results of the analysis are presented in Figure 9.

In Figure 9, it is evident that after the first flow redirection and the following stratified flow region a stable interface is observed. The instability is triggered between positions three and four, respectively, located right before and after the second redirection of the flow where the flow channel is adjusted by the restriction bar. In the subsequent stratified flow domain, an oscillating movement is observed where the individual layers of the polymer and compound are penetrating each other forming a local multilayer structure until the end of the die. For the initial setting of the restriction bar, we could not observe these formations. Hence, the instabilities must be flow induced. The literature provides various possible reasons for the onset of interfacial flow instabilities that might be applicable to our situation such as elongational and compression ratios or forces where the flow is redirected [20]. Additionally, critical interfacial shear stresses [21]; viscosity, shear-rate, and elasticity ratios [22]; and normal stress differences [23] are commonly reported as crucial flow property at the interface. Albeit, literature does not provide a uniform and consistent theory regarding the reasons for interfacial co-extrusion flow instabilities, the onset and appearance will strongly depend on the flow conditions and rheological behavior of the polymers involved. Furthermore, viscoelastic properties are likely to have a significant impact even if the critical conditions can be expressed by representative pure viscous flow properties, and inertia-based turbulences will be highly unlikely due to the very low Reynolds numbers of these types of flows. 

## 5. Conclusions

In this work, we studied the influence of flow conditions on the layer adhesion of co-extruded sheets using a reference system with a polymer and polymer talc compound. We found that for increased extrusion rates the layer adhesion decreases, which is due to decreased contact time. Additionally, interfacial shear stresses are larger for higher extrusion rates which will lead to increased orientation of the polymer chains and according to Kim and Han [9] subsequently cause decreased interdiffusion. Changing the geometry of the flow domain by adjusting the restriction bar, made delamination and hence usage of the roller peel tests to characterize layer adhesion impossible. Inspecting the interface via confocal Raman microscopy revealed a local multi-layer structure of the co-extruded sheet causing superior improvement of layer adhesion. A closer look revealed that interfacial flow instabilities are triggered at the second redirection of the co-extrusion flow, where the restriction bar is located. In the subsequent stratified flow domain, these instabilities are further oscillating and the two layers are locally interpenetrating; then, it is finally stretched to form the multilayer structure resulting in a strong mechanical entanglement of the two layers. 

Commonly, flow instabilities of polymer melt flows and also interfacial co-extrusion flow instabilities are undesired because they lead to optical defects, mechanical weak spots, or layer discontinuities which might be critical for barrier and adhesive layers [24]. The interfacial flow instabilities observed in this study, however, led to considerably improved final mechanical product properties, simultaneously maintaining the two-layer structure and its optical and surface properties. The interfacial strength between two layers is mainly governed by mechanical interlocking, instead of diffusion mechanisms. Nevertheless, for transparent products and transparent cover layers optical properties may be an issue. Moreover, layer continuities and uniformity might be critical, if very thin layers such as barriers and adhesives are involved in the multilayer structure. Especially, if one of the polymers at the interface is an adhesive, layer adhesion is governed mainly by chemical bonds rather than interdiffusion and mechanical entanglements. 

## Figures and Tables

**Table 1:** Melt flow-rate, bulk density and melt density for the polymer and compound.

	MFR (230 °C/2.16 kg) g·10 min^−1^	Solid Density g·cm^−3^	Bulk Density g·cm^−3^	Melt Density g·cm^−3^
polymer	0.30	0.900	0.516	0.745
compound	0.56	1.147	0.763	1.098

**Table 2:** Power-law parameters of the polymer and the compound.

	K Pa·s^n^	n -	$T_{0}$ °C	β K^−1^
polymer	28,658	0.163	240	0.00522
compound	11,373	0.257	240	0.00936

**Table 3:** Temperature settings of extruders, feedblock, and extrusion die.

Zone	Temperature °C
Feed-housing	Water-cooled
Extruder Zone 1	210
Extruder Zone 2	230
Extruder Zone 3	220
Adapter	210
Feedblock	210
Extrusion die	210

**Table 4:** Variation of screw speed N and back-pressure p for screw characterization.

Parameter	Unit	Min	Max	Divisions
Screw speed N	rpm	10	150	7
Back-pressure p	bar	25	300	11

**Table 5:** Rounded values of the constants for ${\dot{m}}_{polymer}=f\left(N,p\right)$.

Constant	Value	Constant	Value	Constant	Value
$a_{0}$	0.010427	$a_{3}$	−1.3905 × 10^−4^	$a_{6}$	0.0019322
$a_{1}$	1.0510 × 10^−4^	$a_{4}$	0.0028343	$a_{7}$	−0.0025606
$a_{2}$	0.050902	$a_{5}$	−0.0026408	$a_{8}$	0.073228

**Table 6:** Rounded values of the constants for ${\dot{m}}_{compound}=f\left(N,p\right)$.

Constant	Value	Constant	Value	Constant	Value
$b_{0}$	−0.056897	$b_{3}$	2.5372 × 10^−4^	$b_{6}$	4.4212 × 10^−14^
$b_{1}$	0.18002	$b_{4}$	5.4871 × 10^−7^	$b_{7}$	−1.2538 × 10^−11^
$b_{2}$	−2.4022 × 10^−4^	$b_{5}$	1.7442 × 10^−14^		

**Table 7:** Error analysis of the symbolic regression models.

Parameter	Unit	Polymer	Compound
$\mathrm{Pearson}\;R^{2}$	-	0.9979	0.9988
Mean absolute error MAE	kg/h	0.1049	0.1545
Mean relative error MRE	%	2.303	2.475

**Table 8:** Processing conditions: individual throughputs, overall throughputs, channel height in the restricted flow area, mean residence time within the die for co-extrusion, melt temperature of the polymer $T_{melt,polymer}$, and melt temperature of the compound $T_{melt,compound}$ of samples 1 to 6 out of polymer and compound.

**Sample**	${\dot{m}}_{polymer}$ kg·h^−1^	${\dot{m}}_{compound}$ kg·h^−1^	${\dot{m}}_{total}$ kg·h^−1^	$h_{restrict}$ mm	$\bar{t}$ s	$T_{melt,polymer}$ °C	$T_{melt,compound}$ °C
1	2.3	9.2	11.5	1.6	18.0	242	241
2	3.1	12.3	15.4		13.4	242	241
3	4.0	16.0	20.0		10.4	241	244
4	2.3	9.2	11.5	1.2	17.2	242	241
5	3.1	12.3	15.4		12.8	242	241
6	4.0	16.0	20.0		9.9	241	244

## Data Availability

The data presented in this study are available on request from the corresponding author.
